# Public Perspectives on Lifestyle-Related Behavior Change for Dementia Risk Reduction: An Exploratory Qualitative Study in The Netherlands

**DOI:** 10.3233/JAD-230217

**Published:** 2023-10-10

**Authors:** Jeroen Bruinsma, Irene Heger, Vasileios S. Loukas, Thomas Kassiotis, Georgia Karanasiou, Dimitrios I. Fotiadis, Sten Hanke, Rik Crutzen

**Affiliations:** a Department of Health Promotion of the Care and Public Health Research Institute at Maastricht University, Maastricht, The Netherlands; b Department of Psychiatry and Neuropsychology of the School for Mental Health and Neuroscience at Maastricht University, Maastricht, The Netherlands; c Unit of Medical Technology and Intelligent Information Systems, Department of Materials Science and Engineering, University of Ioannina, Ioannina, Greece; d Biomedical Research Institute, Foundation for Research and Technology - Hellas, FORTH-BRI, Ioannina, Greece; e Computational BioMedicine Laboratory, Institute of Computer Science, Foundation for Research and Technology – Hellas, FORTH-ICS-CBML, Heraklion, Crete, Greece; f Institute of eHealth at University of Applied Science at FH Joanneum, Graz, Austria

**Keywords:** Alzheimer’s disease, behavior change, dementia, health promotion, prevention, public health

## Abstract

**Background:**

There is accumulating evidence that addressing modifiable risk and protective factors has an impact on dementia rates. Insight into the public’s perspectives on dementia risk reduction is needed to inform future individual-level interventions and public health approaches.

**Objective:**

This study explores the publics’ openness towards dementia risk reduction and willingness towards changing lifestyle behavior to reduce the future risk for dementia.

**Methods:**

Using a screening questionnaire, participants were purposively selected based on lifestyle behaviors that are associated with dementia risk. One-on-one interviews were used to explore their openness towards dementia risk reduction and willingness towards behavior change. Independently, two researchers performed an inductive content analysis.

**Results:**

Interviews were conducted with 23 participants aged from 40 to 79 years. Main themes that were identified from the data were: 1) abstractness of dementia risk reduction, 2) ambivalence towards changing behavior, 3) negative self-image and low behavioral control, and 4) all-or-nothing thinking about lifestyle change.

**Conclusions:**

The concept of dementia risk reduction seems difficult to translate to the personal context, particularly if individuals perceive that dementia would occur decades in the future. This is problematic because a large proportion of the public needs a healthier lifestyle to reduce the incidence of dementia. Translating healthy intentions into behavior is complex and involves overcoming a variety of barriers that complicate dementia risk reduction initiatives. Support is needed for individuals who experience additional obstacles that obstruct commencing to a healthier lifestyle (e.g., negative self-image, engaging in multiple unhealthy behaviors, unrealistic perceptions about lifestyle change).

## INTRODUCTION

In recent years, increased attention has been paid to the promotion healthy lifestyle behavior and cardiovascular risk management to reduce the expected increase of dementia cases [1, 2]. The current preventive strategy builds upon epidemiological evidence regarding modifiable protective and risk factors that underly cognitive decline and dementia. It is estimated that a substantial part of the global dementia cases are related to various lifestyle-related behaviors [3, 4]. Examples of protective behaviors are a healthy diet, physical activity, smoking cessation, low to moderate alcohol consumption, and engagement in social and cognitive activities [5, 6]. Openness towards dementia risk reduction is attributed to personal experiences with dementia, awareness about risks, and health beliefs [7, 8]. Several barriers that complicate lifestyle change for dementia risk reduction have already been identified, such as time restraints or skepticism towards the relevance and effectiveness of risk reduction [8].

More insight in the perspectives of people from the general public about dementia risk reduction is needed to inform future individual-level behavior change interventions and population-based health approaches [9, 10]. Therefore, this study explores the Dutch public’s openness towards dementia risk reduction as well as the willingness towards altering lifestyle behaviors in view of future dementia risk reduction. In the short run, the findings of this study are used to guide the development of intervention materials and strategies as part of the European LETHE-project. LETHE focusses on individualized risk prediction and digitally assisted lifestyle interventions to reduce the risk for dementia. In the long run, the findings offer insights for the National Dementia Strategy of the Dutch Ministry of Health, which emphasizes lifestyle prevention as a key approach [11]. For instance, the findings can inform communication strategies to raise awareness about dementia risk reduction, guide policy endeavors to make lifestyle change more accessible, or direct efforts to offer lifestyle intervention.

## METHODS

This qualitative study used a screening questionnaire to select participants for one-on-one interviews about dementia risk reduction and lifestyle change. The findings are reported using the Consolidated criteria for Reporting Qualitative research checklist (Supplementary Table 1) [12]. All research materials are openly accessible through the Open Science Framework to allow scrutiny, foster accurate replication, and enable future data synthesis [13].

### Recruitment

Between January 2022 and April 2022, participants aged 40 to 79 years old were recruited for individual interviews, as associations between lifestyle and dementia are well-established in mid-life and late-life [5, 14, 15]. Participants were recruited in the South-Limburg area of the Netherlands by spreading posters. These posters were displayed near the entrance or in waiting rooms of local health centers, pharmacies, supermarkets, and in the Maastricht University Medical Centre (MUMC+). The posters were also shared via social media (i.e., LinkedIn and Facebook). On the poster (Supplementary Figure 1), participants were asked if they engaged in unhealthy eating, physical inactivity, alcohol consumption or smoking, while inviting them to join the conversation about lifestyle. The poster did not contain information about dementia risk reduction to solely include participants based upon lifestyle behavior.

**Fig. 1:**
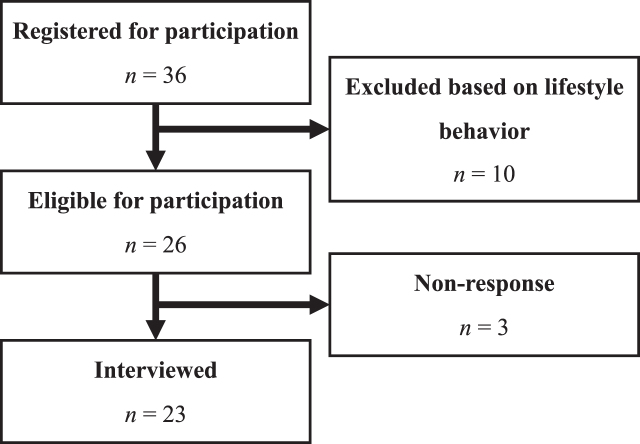
Recruitment and inclusion.

Participants registered through a screening questionnaire that was created in Form Desk. The screening questionnaire (Supplementary File 1) was based on the validated LIfestyle for BRAin health (LIBRA) index and asked participants to self-report twelve protective and risk factors for dementia [5, 16]. Using purposeful sampling [17, 18], 23 participants were selected based on the absence of protective behavioral factors and/or the presence of behavioral risk factors associated with dementia. Participants were eligible for inclusion if they self-reported at least one lifestyle-related behavior, included in the LIBRA (Fig. 1), that could be improved to reduce future dementia risk. In the selected sample, physical inactivity was the most prevalent selection criterion, followed by high alcohol consumption, non-adherence to the Mediterranean diet, smoking, and low levels of cognitive activity (Table 1). Most of the participants were female (*n* = 15). The mean age of the participants was 58.1 years (SD = 9.9) and ranged from 40 to 79 years.

**Table 1:** The LIfestyle for BRAin health (LIBRA) index [6, 16] (*n* = 23)

Protective factors	*n*
**No or low-moderate alcohol consumption**	11
**Mediterranean diet**	14
**High cognitive activity**	19
**Risk factors**	*n*
**Physical inactivity**	13
**Smoking**	5
Coronary heart disease	3
Renal disfunction	0
Diabetes	0
High cholesterol	8
Obesity (Body Mass Index ≥30)	8
Hypertension	8
Depression	1

**In bold** the behaviors that were used to select participants. These behaviors were self-reported.

### Data collection

One-on-one interviews of approximately 40-min duration were conducted at the home of the participants or online via Microsoft Teams. This was based on the preference of the participants as social restrictions were in place to prevent the spread of COVID-19. The ability to participate online allowed persons who resided outside the South-Limburg region to participate.

All interviews were moderated by a male postdoctoral researcher (JB) with a background in health sciences who is experienced in conducting, analyzing, and reporting qualitative research.

A semi-structured conversation tool (Supplementary File 2) guided the interviews and was based on psychological construct definitions and measurement instructions [19]. The initial part of the interview explored general lifestyle-related behavior. This was followed by a reflection on the participants’ responses on the screening questionnaire and by subsequent questions about the openness of changing certain lifestyle behaviors to reduce the future risk for dementia.

### Data analysis

Audio-recordings of the interviews and fieldnotes were transcribed verbatim. Two authors (JB and IH) independently performed an inductive content analysis by open coding in Atlas.ti [20]. This approach allowed the exploration of concepts derived directly from the data, without relying on predetermined categories. As a result, it enabled the identification of new patterns and themes that influence the openness towards dementia risk reduction as well as the willingness towards altering lifestyle behavior in view of future dementia risk reduction. During weekly discussion sessions, axial and selective coding were performed to reach consensus about the codes and to link important themes together [21]. Thematic data saturation was verified by comparing if newly analyzed interviews generated less than 5% new thematic codes [22, 23] (Supplementary Table 2). Main themes that were generated based on the data were discussed with the last author (RC) to yield the key results. Transcripts were not returned to participants, but instead the key results were discussed with three randomly selected participants via member reflections using individual think-aloud sessions that were structured with PowerPoint slides containing the findings (Supplementary File 3) [24].

### Trustworthiness

Methods to achieve triangulation were embedded to increase the credibility of findings. Multimethod triangulation was achieved by collecting data via a screening questionnaire, the interview, and member reflections [25]. Investigator triangulation was achieved by involving two researchers who independently coded the data, reached consensus via discussion, and by successively discussing the findings with the last author and the participants [26]. To allow insight in our data analysis process all the research materials are openly available, including the coding tree (Supplementary File 4).

### Ethics

The study protocol was approved by the Research Ethics Committee of the Faculty of Health, Medicine and Life Sciences of Maastricht University, the Netherlands (FHML-REC/2021/105). Participants were phoned after registration, received an information letter via email, and gave written consent before participating in the interview. Data of persons who were not interviewed were deleted.

## RESULTS

Four themes were identified from the data, namely 1) abstractness of dementia risk reduction, 2) ambivalence towards changing behavior, 3) negative self-image and low behavioral control, and 4) all-or-nothing thinking about lifestyle change.

### Abstractness of dementia risk reduction

Generally, participants were positive about initiatives striving for the prevention of dementia in individuals at risk. This was especially the case if participants were acquainted with persons with dementia, for example they had family members with dementia or worked in healthcare. To varying degrees, many expressed being aware of the relationship between lifestyle and dementia, and occasionally media appearances around the topic were addressed by the participants. Only few expressed skepticisms about the effectiveness of a healthy lifestyle or perceived that genetics were a decisive factor for developing dementia. Yet, for most participants it remained difficult to conceptualize to what extent their lifestyle contributed their personal risk for developing dementia in the future. Also, most expressed the desire to know how certain lifestyle improvements would decrease their personal risk for dementia. Partly, the abstractness of personal risk was attributed to the feeling that dementia occurred decades away in the future, this was especially perceived by participants in midlife. Additionally, most felt uncertain about the effect of lifestyle change because successful prevention could not be guaranteed.

“*Prevention is better than a cure. However, dementia remains far away in the future*” – *ID35*

“*I would be prepared to change my lifestyle if it is guaranteed to lower my personal risk for dementia.*” – *ID17*

### Ambivalence towards changing behavior

Most participants perceived room for improvement and expressed intentions towards lifestyle change. These positive intentions were attributed to various short-term benefits (e.g., it gives positive energy) and long-term benefits (e.g., healthy aging and staying independent). When the interviews narrowed down on discussing specific behavior change to reduce future dementia risk, conflicting feelings about lifestyle change were observed. Despite intentions towards a (general) healthier lifestyle, participants were more resistant to change specific behaviors. To illustrate, they mitigated the negative effects of specific unhealthy behavior, suggested to compensate for it, or stressed they had already made positive lifestyle adaptations. A reoccurring conversation theme was that participants felt an immediate health threat was needed to initiate lifestyle change. However, it was observed that some had already experienced a health threatening event but still had the need for a ‘wake-up call’.

*One of my friends recently found out she had clogged arteries. I imagine if you hear about this you would act.* – *ID09*

*If I get complaints such as heart issues. So, a real warning. Then I would say* ‘*I*’*m done smoking*’. *[... ] In 2005 I had a light heart attack.* – *ID21*

### Negative self-image and low behavioral control

Participants’ doubts about implementing sustainable behavior change were attributed to having a negative self-image. As a consequence of previous attempts to change, some participants felt they had limited control over lifestyle-related behavior. Occasionally, this resulted in negative self-image. To illustrate, some participants felt they lacked willpower and perceived themselves as lazy, weak, or incapable. The opposite was also observed as participants perceived that successful lifestyle change would result in more self-appreciation. Especially, participants with a negative self-image perceived difficulty prioritizing self-care and experienced more psychological and environmental obstacles that complicated changing to a healthier lifestyle (e.g., time restraints or social pressure). It was observed that participants with negative self-image more often discussed socioeconomic difficulty such as unemployment, financial problems, living alone or living in social housing.

“*It is confronting that I*’*m too heavy. I will feel better about myself if I lose weight. This has to do with self-control.*” – *ID39*

“*You need self-worth to say to yourself* ‘*Let*’*s cook a nice dinner for myself*’*. I just eat because I must.*” – *ID02*

### All-or-nothing thinking about lifestyle change

Generally, participants applied all-or-nothing thinking towards goal setting and lifestyle-related behavior change. Participants frequently perceived that drastic lifestyle change was needed and felt they had to ‘flip a switch’ to find the right mindset to achieve this. Occasionally, this fact was attributed to lacking knowledge about lifestyle guidelines, for example about healthy diet, alcohol consumption or physical activity. Although many perceived that radical lifestyle change, as discussed in the paragraph “Ambivalence towards changing behavior”, was the key to success, most had no specific or realistic goals. To exemplify, participants suggested to never eat chocolate again while they currently (over)consumed it daily or thought about running multiple times a week to participate in running events while they had not exercised in years. Having an overly ambitious goal towards lifestyle change without having a realistic plan of action showed to obstruct the process of making lifestyle changes.

“*I just need to do it. But that*’*s what I*’*ve been trying for ages.*” – *ID14*

“*The idea that I must stop eating chocolate is terrible. It is such an inner struggle, so I*’*m not going to do it.*” – *ID08*

### Results of the member reflections

The three randomly selected participants largely verified our findings during the think-aloud sessions, where they reflected on the key findings. To illustrate, they confirmed that dementia risk was difficult to interpret for them personally and felt it was complex to imagine the long-term effects of change in lifestyle behavior. All participants acknowledged having (generic) healthy lifestyle intentions but had no (specific) goals to change behavior and all stressed the importance of adequate goal setting. Although none of these participants perceived having a negative self-image, they were able to envision how this adversely affected the ability to change lifestyle behavior. To nuance our findings, they suggested stressing the importance of personal coaching for individuals with a negative self-image and low perceived behavioral control.

## DISCUSSION

This qualitative study investigated the publics’ openness towards dementia risk reduction and willingness towards changing lifestyle behavior to reduce the future risk for dementia. In general, the Dutch public is positive about the concept of dementia risk reduction, particularly if they had family members with dementia or worked in healthcare [7, 8]. Many of our participants were able to identify factors that are associated with a (brain) healthy lifestyle and perceived room to improve their own lifestyle. Partly, this stemmed from recent media attention in the Netherlands highlighting the importance of a healthy lifestyle to decrease the future risk for dementia. These findings are positive because earlier studies indicate a lack of public awareness about dementia risk reduction [27], also in the Limburg region of the Netherlands [28] where our study was conducted. The results illustrate that awareness about dementia risk reduction can generate a window of opportunity for health promotion. Nonetheless, the exhibited findings also demonstrate the complexity of achieving actual behavior change. More specifically, personal risks for dementia were perceived as abstract and it appeared difficult for participants to conceptualize the long-term benefits of lifestyle change. This made it challenging to translate the concept of dementia risk reduction into the personal context. Particularly, for middle-aged individuals it may feel that dementia will occur decades in the future [29]. In turn, they may feel less susceptible and therefore less inclined to act. This is problematic because a large proportion of the Dutch adults is engaging in lifestyle behaviors that are associated to higher dementia risk. In turn, a large proportion of the public needs to adopt a healthier lifestyle to reduce the expected increase of dementia cases [29, 30]. To improve the health of the public, the Dutch government recently released a prevention agreement to reduce unhealthy lifestyle behaviors before 2040. Specifically, the aim is to decrease smoking figures from 19% to 5%, overweight from 50% to 38%, and excessive consumption of alcohol from 8.5% to 5% [31, 32]. Achieving these aims is a challenge, as our findings show that changing lifestyle behavior is a complex multi-faceted process that involves overcoming a variety of psychological and environmental barriers [33]. Our findings illustrate some of the barriers that complicate translating (generic) healthy intentions to (specific) behavior change. Healthy intentions are not necessarily intrinsically motivated, and it is well-established that discrepancy between intention and behavior is more pronounced for extrinsically motivated behavior [34]. Although extrinsic motivation can initiate lifestyle change, intrinsic motivation increases the likelihood of maintenance of behavior after change [35, 36]. To initiate sustainable lifestyle change, dementia risk reduction initiatives should encourage people to think about personal relevant short-term benefits of healthy behavior. Our findings indicate that specific guidance is needed for individuals with a negative self-image and low perceived behavioral control because they experience additional barriers that obstruct commencing a healthier lifestyle. Often these feelings were the result of failed attempts to implement lifestyle improvements. Similar feelings may be experienced by participants who drop out of dementia risk reduction interventions. Therefore, a direction for support is to help individuals who struggle with maintenance of behavior changes in reattributing experienced relapse and encourage them to learn from the experience by seeing it as part of the change process they go through[37, 38].

Although no data about socioeconomic position was purposively collected via the screening questionnaire, it was observed during the qualitative analysis that a negative self-image was more common in participants with a low socioeconomic position. For instance, these participants discussed more often unemployment or resided in social housing. It is known that socioeconomic deprivation is negatively related to self-concept [39], lifestyle-related inequalities in health-behavior [40], and it explains differences in modifiable risk factors for dementia [41]. A particular limitation of individual-level interventions aiming for health promotion is that they often reach healthier participants with a higher socioeconomic position. These individuals also seem to benefit more from participation in interventions because having a (relatively) healthy lifestyle requires smaller adaptation and a good socioeconomic position is accompanied by having more resources to accomplish lifestyle change [8, 42]. Therefore, interventions aiming for dementia risk reduction should specifically put effort in including and supporting participants with multiple unhealthy behaviors and a lower socioeconomic position. To accomplish adequate guidance, interventions should incorporate methods to guide goal setting and action planning, as this seems to strengthen the self-concept, increase self-efficacy, create confidence, and this will help in overcoming psychological and environmental barriers [33, 43]. To illustrate, methods to guide goal setting and action planning are setting achievable tasks that gradually increase in difficulty level, offering guided practice and coach supervision, planning coping responses to overcome barriers and manage relapse, and monitoring and appraising (small) achievements via feedback[33].

In the context of dementia prevention, most research has focused on mapping epidemiological risk factors and developing individual-level interventions for high-risk populations [30]. It is questionable if this approach can effectively reduce the high incidence of new dementia cases because dementia and its risk factors are highly prevalent on population level. In contrast to individual-level interventions, population-based approaches try to promote healthy lifestyle behavior to the entire society by making it more accessible and convenient [44, 45]. For instance, in the Netherlands, the government strives to improve public health by excise tax on unhealthy products (e.g., cigarettes, alcohol or sugar) or by providing sufficient and appropriate (outdoor) sports facilities (e.g., parks and gyms) [32]. A good balance between these population-based approaches and individual-level interventions is needed as this will allow health improvement of all individuals in a society and offer additional support to those who need it most [30].

### Considerations

This qualitative study aimed to obtain insight into the extent to which people are open and willing to change lifestyle-related behavior to reduce the future risk for dementia. It succeeded in identifying specific challenges that complicate behavior change for dementia risk reduction. The present study embedded various methods to increase the credibility of findings, such as triangulation and member reflections. Although the findings provide a unique insight in the perceptions of Dutch persons in mid-life and late-life, future research is warranted to distinguish the relative importance of the various perceptions in both the Netherlands and an international context. Therefore, a questionnaire study is underway in the Netherlands [46]. This study will also provide further insight into specific behavioral determinants that underly the willingness to change lifestyle-related behaviors that are relevant in the context of dementia risk reduction.

### Conclusions and directions for the future

Lifestyle-related behavior change for dementia risk reduction is extremely challenging. It is complex for people to interpret personal dementia risk and to translate this into specific behavior change. Especially individuals with a negative self-image and low perceived behavioral control experience barriers that complicate commencing towards a healthier lifestyle. Helping them to formulate specific goals and realistic plans of action may guide them towards a brain healthier lifestyle. Given the complexity of lifestyle-related behavior change, it is required to collaborate with population-based health promotion initiatives to reduce the increasing number of dementia cases.

## Supplementary Material

Supplementary Material

## Data Availability

The data supporting the findings is available via supplementary materials and via the Open Science Framework via https://doi.org/10.17605/OSF.IO/GB4M5.
