# Supplementary material for: Public Perspectives on Lifestyle-Related Behavior Change for Dementia Risk Reduction: An Exploratory Qualitative Study in The Netherlands
Source: J Alzheimers Dis. 2023 Oct 10;95(4):1635–42. doi: 10.3233/JAD-230217 (PMC10578282; doi:10.3233/JAD-230217)
Supplement: Supplementary Material [file jad-95-jad230217-s001.pdf]

Supplementary Material

The public's perspectives on lifestyle-related behavior change for dementia risk reduction.

Supplementary Table 1. COREQ checklist

COREQ (Consolidated criteria for Reporting Qualitative research) Checklist

A checklist of items that should be included in reports of qualitative research. You must report the page number in your manuscript where you consider each of the items listed in this checklist. If you have not included this information, either revise your manuscript accordingly before submitting or note N/A.

**No. Item**	**Guide questions/description**	**Reported on Page #**
**Domain 1: Research team and reflexivity**		
Personal Characteristics		
1. Interviewer/facilitator	Which author/s conducted the interview or focus group?	Page 5 / Data collection
2. Credentials	What were the researcher's credentials? e.g. PhD, MD	Page 5 / Data collection
3. Occupation	What was their occupation at the time of the study?	Page 5 / Data collection
4. Gender	Was the researcher male or female?	Page 5 / Data collection
5. Experience and training	What experience or training did the researcher have?	Page 5 / Data collection
Relationship with participants		
6. Relationship established	Was a relationship established prior to study commencement?	Page 4 / Recruitment
7. Participant knowledge of the interviewer	What did the participants know about the researcher? e.g. personal goals, reasons for doing the research	Page 4 / Recruitment
8. Interviewer characteristics	What characteristics were reported about the interviewer/facilitator? e.g. Bias, assumptions, reasons and interests in the research topic	Page 4 / Recruitment<br>Page 5 / Data collection

**Domain 2: study design**		
Theoretical framework		
9. Methodological orientation and Theory	What methodological orientation was stated to underpin the study? e.g. grounded theory, discourse analysis, ethnography, phenomenology, content analysis	Page 6 / Data-analysis
Participant selection		
10. Sampling	How were participants selected? e.g. purposive, convenience, consecutive, snowball	Page 4 / Recruitment
11. Method of approach	How were participants approached? e.g. face-to-face, telephone, mail, email	Page 7 / Ethics
12. Sample size	How many participants were in the study?	Page 4 / Recruitment
13. Non-participation	How many people refused to participate or dropped out? Reasons?	Page 4 / Recruitment
Setting		
14. Setting of data collection	Where was the data collected? e.g. home, clinic, workplace	Page 5 / Data collection
15. Presence of non-participants	Was anyone else present besides the participants and researchers?	Page 5 / Data collection
16. Description of sample	What are the important characteristics of the sample? e.g. demographic data, date	Page 4 / Recruitment
Data collection		
17. Interview guide	Were questions, prompts, guides provided by the authors? Was it pilot tested?	Page 5 / Data collection
18. Repeat interviews	Were repeat interviews carried out? If yes, how many?	Page 6 / Trustworthiness
19. Audio/visual recording	Did the research use audio or visual recording to collect the data?	Page 6 / Data-analysis
20. Field notes	Were field notes made during and/or after the interview or focus group?	Page 6 / Data-analysis
21. Duration	What was the duration of the interviews or focus group?	Page 5 / Data collection
22. Data saturation	Was data saturation discussed?	Page 6 / Data-analysis
23. Transcripts returned	Were transcripts returned to participants for comment and/or correction?	Page 6 / Trustworthiness
**Domain 3: analysis and findings**		

Data analysis		
24. Number of data coders	How many data coders coded the data?	Page 6 / Data-analysis
25. Description of the coding tree	Did authors provide a description of the coding tree?	Supplementary Files
26. Derivation of themes	Were themes identified in advance or derived from the data?	Page 6 / Data-analysis
27. Software	What software, if applicable, was used to manage the data?	Page 6 / Data-analysis
28. Participant checking	Did participants provide feedback on the findings?	Page 6 / Trustworthiness Page 9 / Member reflections
Reporting		
29. Quotations presented	Were participant quotations presented to illustrate the themes/findings? Was each quotation identified? e.g. participant number	Page 7 / Results
30. Data and findings consistent	Was there consistency between the data presented and the findings?	Page 7 / Results
31. Clarity of major themes	Were major themes clearly presented in the findings?	Page 7 / Results
32. Clarity of minor themes	Is there a description of diverse cases or discussion of minor themes?	Page 7 / Results

Developed from: Tong A, Sainsbury P, Craig J. Consolidated criteria for reporting qualitative research (COREQ): a 32-item checklist for interviews and focus groups. *International Journal of Quality in Health Care.* 2007. Volume 19, Number 6: pp. 349-357

Once you have completed this checklist, please save a copy and upload it as part of your submission. DO NOT include this checklist as part of the main manuscript document. It must be uploaded as a separate file.

Supplementary Figure 1. Poster used to recruit participants

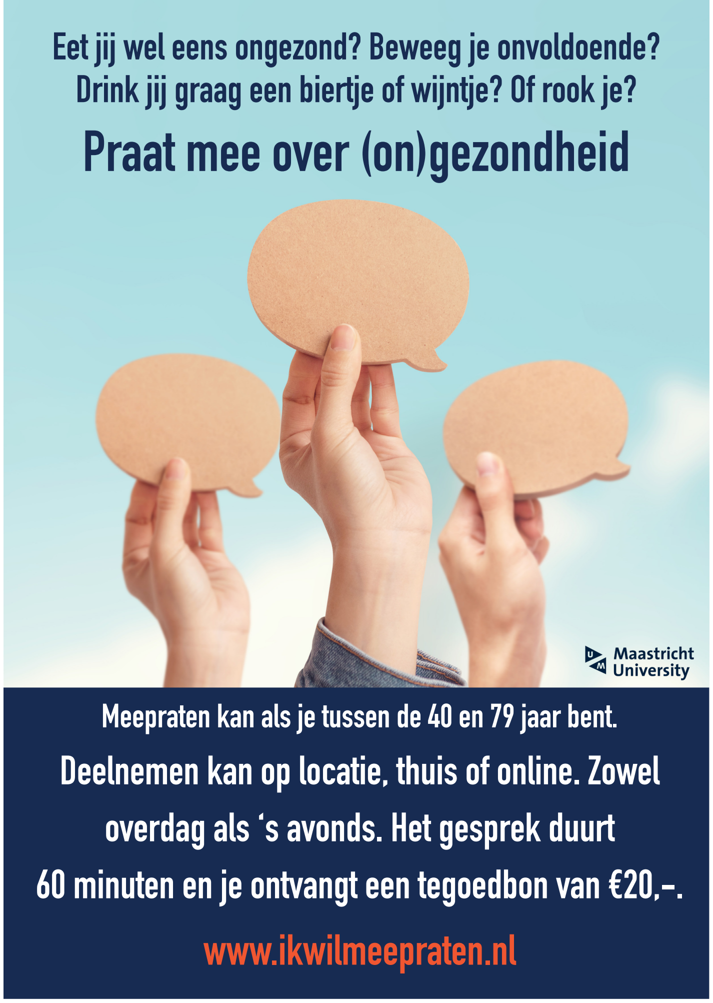

English translation of text

Do you eat unhealthy? Are you physically inactive?
Do you like to drink beer or wine? Or do you smoke?
Let's talk about (un)healthy lifestyle.

You can join the conversation is you are aged between 40 and 79 years.
You can participate in an interview on location, from home or online, during the day or in the evening.
The interview lasts around 1 hour and you will receive a gift card of €20,-.
www.ikwilmeepraten.nl

Supplementary File 1. Screening questionnaire (followed by an English Translation)

Registratieformulier deelname aan wetenschappelijk onderzoek

Onderzoek: Leefstijl en dementie

Wij vragen u dit registratieformulier in te vullen voor deelname aan het onderzoek leefstijl en hersengezondheid. Op dit registratieformulier vragen wij enkele persoonlijke gegevens van u. Bijvoorbeeld uw telefoonnummer, geslacht, leeftijd en levensstijl. Wij bekijken aan de hand van deze gegevens of uw wordt uitgenodigd voor een interview. Wij nemen hierover binnen enkele weken contact met u op. Als u niet wordt uitgenodigd voor een interview krijgt u daarover bericht en worden uw persoonlijk gegevens vernietigt. Als u deelneemt aan het interview worden uw persoonlijke gegevens opgeslagen. Vooraf aan het interview ontvangt u daarover meer informatie en geeft u apart toestemming.

Alvast bedankt voor uw deelname. Als u vragen heeft laat het ons gerust weten! U kunt contact met ons opnemen via: [emailadres onderzoekers]

Hoe oud bent u?

[Antwoord: in jaren]

Wat is uw geslacht?

[Antwoord: Mannelijk – Vrouwelijk – Anders]

Wat is uw lengte?

[Antwoord: in cm range van 120 tot 220cm]

Wat is uw gewicht?

[Antwoord: in kg range van 45 tot 180kg]

Rookt u?

[Antwoord: Ja – Nee]

Hoe vaak drinkt u alcoholhoudende drank?

nooit (geen vervolgvragen)

maandelijks of minder (geen vervolgvragen)

2 tot 4 keer per maand

2 tot 3 keer per week

4 of meer keer per week

[Vervolgvraag bij 2 of 4 keer per maand of vaker]

Hoeveel glazen alcohol drinkt u op een typische dag wanneer u drinkt?

1 tot 2

3 tot 4

5 tot 6

7 tot 9

10 of meer

Heeft uw arts u ooit verteld dat u … hebt

	**Ja**	**Nee**	**Weet ik niet**
**Een depressie**			
**Een hoog cholesterol**			
**Een chronische nierziekte**			
**Een hoge bloeddruk**			
**Suikerziekte**			
**Een aandoening aan hart of bloedvaten**			

Bent u opgewekt en vindt u het leven de moeite waard?

[Antwoord: Ja – Nee – Een beetje]

Bent u een actief persoon die regelmatig aan beweging doet waarbij u licht zweet?

[Antwoord: Ja – Nee – Een beetje]

Bent u leergierig? Bijvoorbeeld omdat u nieuwe dingen leert, muziek maakt, of puzzelt?

[Antwoord: Ja – Nee – Een beetje]

Bent u een persoon die gezond eet?

[Antwoord: Ja – Nee – Een beetje]

Eet u wekelijks vis, groenten, peulvruchten, (olijf)olie of pasta eet. Ook wel bekend als een "mediterraan dieet".

[Antwoord: Ja – Nee – Een beetje]

<u>Contactgegevens voor het inplannen van een interview</u>

Hoe heet u?

[Antwoord: vrije tekst]

Waar woont u?

[Antwoord: vrije tekst]

Wat is uw telefoonnummer?

[Antwoord: vrije tekst]

Wat is uw emailadres?

[Antwoord: vrije tekst]

Registration form for participating in research

Study: Lifestyle and dementia

We ask you to complete this registration form in order to participate in our study on lifestyle and brain health. In this registration form we ask you to provide some personal contact details, such as your phone number, gender, age and lifestyle. Based on your registration we will verify if you are able to participate in the interview. We will contact you about this within a couple of weeks. If you are not invited for the interview, we will contact you and your personal contact details will be deleted. In case you are eligible for participation in the interview we will store your personal contact details. In advance of the interview, you will receive more information via an information letter and sign an informed consent form.

We appreciate your willingness for participation. Feel free to contact us in case you have any questions. You can contact us via: [emailadress researchers]

What is your age?

[in years]

What is your gender?

[Male – Female – Other]

What is your length?

[in cm, ranging from 120 to 220cm]

What is your weight?

[in kg ranging from 45 to 180kg]

Do you smoke?

[Yes – No]

How often do you consume alcohol?

never (no follow-up question on alcohol)

monthly or less (no follow-up question on alcohol)

2 to 4 times a month

2 to 3 times a week

4 or more times a week

[follow-up question is provided of participants consume alcohol 2 to 4 times a month or more]

How many glasses of alcohol do you consume on a typical occasion?

1 to 2

3 to 4

5 to 6

7 to 9

10 or more

Did your doctor ever told you that you have…

	**Ja**	**Nee**	**Weet ik niet**
**A depression**			
**High cholesterol**			
**Chronic kidney disease**			
**High blood pressure**			
**Diabetes**			
**Cardiovascular disease**			

Do you feel cheerful, and do you think life is worth living?

[Yes – No – A little]

Are you a person who is regularly physically active that makes you sweat lightly?

[Yes – No – A little]

Are you eager to learn new things? For example, how to play music or making puzzles?

[Yes – No – A little]

Are you a person with a healthy diet?

[Yes – No – A little]

Do you eat fish, vegetables, legumes, (olive) oil or pasta on a weekly basis? Also known as a "Mediterranean diet".

[Yes – No – A little]

Contact details required to plan the interview

Name:

[Free text]

Where do you live?

[Free text]

What is your phone number?

[Free text]

What is your email address?

[Free text]

Supplementary File 2. Interview guide in Dutch (followed by an English Translation)

Onderzoek: Leefstijl en dementie

Introductie

In een leven vol verleidingen en drukke agenda's is het soms lastig om gezond te leven. Met dit onderzoek brengen we in kaart wat beweegredenen zijn om wel of niet uw gedrag te veranderen om uw hersengezondheid te verbeteren en de kans op dementie te verminderen.

Tijdens dit gesprek van 60 minuten, stel ik u een aantal vragen over uw levensstijl. In het eerste deel bespreken we wat het voor u nou eigenlijk betekent om gezond te leven. En wat voor u beweegredenen zijn om wel of niet uw levensstijl te veranderen. Wij zijn geïnteresseerd in uw mening en u kunt dus geen goede of foute antwoorden geven.

In het tweede deel kijken we samen naar uw antwoorden op het registratieformulier dat u heeft ingevuld. We bespreken gedrag dat belangrijk is voor uw hersengezondheid.

Dit gesprek is onderdeel van een wetenschappelijk onderzoek. Daarom wil ik graag een aantal randvoorwaarden met u bespreken.

Dit gesprek nemen we op zodat we een zorgvuldige analyse kunnen verrichten. Graag zou ik de audio-recorder nu aanzetten en samen met u willen kijken naar uw rechten tijdens dit onderzoek.

Een aantal weken geleden heeft u een informatiebrief gekregen. U heeft toestemming gegeven tot deelname aan dit onderzoek. Graag wil ik beklemtonen dat uw deelname vrijblijvend is. U mag ten alle tijden stoppen. Ook tijdens dit gesprek. U hoeft daar geen reden voor op te geven.

Als u tijdens het gesprek iets vertelt en liever niet heeft dat wij dat gebruiken dan kunt u dat gerust aangeven. Dat mag ook na afloop.

1. Dit interview nemen we op via een audio-recorder. **Vindt u dat oké?**

2. In anonieme vorm gebruiken we dit interview om een wetenschappelijke rapportage te schrijven. Die is openbaar toegankelijk. **Heeft u daar bezwaar tegen?**

3. Dit gesprek zullen we woord voor woord uittypen. Gegevens die tot u te herleiden zijn worden vervangen door een code. Zo vervangen we uw woonplaats bijvoorbeeld door de code 'woonplaats'. Om andere wetenschappers in de gelegenheid te stellen te leren van onze onderzoeksresultaten stellen wij de uitgetypte interviews op aanvraag beschikbaar. U heeft op het toestemmingsformulier aangegeven dat u daar **wel/niet** akkoord mee gaat. **Kunt u dat bevestigen?**

Heeft u op dit moment nog vragen? Dan stel ik voor dat we gaan beginnen.

Deel 1: Algemene perspectieven over levensstijl

1. Wat verstaat u eigenlijk onder een gezonde levensstijl?

Doorvragen:

1. Heeft u het idee dat u op dit moment gezond leeft? Kunt u uitleggen waarom wel/niet?
2. Wat is volgens u de meerwaarde van een gezonde levensstijl?
    a. Welke meerwaarde heeft een gezonde levensstijl op de lang termijn?
    b. Zijn er ook korte termijneffecten?

2. Wat kunt u op dit moment doen om gezonder te leven?

Doorvragen:

1. U noemt een aantal dingen op. Wat is voor u het belangrijkste? Waarom?
    a. Stel u zou … wat verwacht u dan dat er gebeurt? (Outcome expectancy)
    b. Doorvragen lang- en korte termijneffecten.

3. Wist u dat een gezonde levensstijl goed is voor uw hersengezondheid en het risico op dementie kan verminderen? (Knowledge)

Doorvragen:

Uit onderzoek blijkt dat de kans op dementie ongeveer 30-40% lager is bij mensen die niet roken, genoeg bewegen, weinig alcohol drinken, gezond eten, sociaal actief en leergierig zijn.

1. Wat vindt u hiervan? (Attitude)
2. Leeft u momenteel gezond genoeg om uw risico op dementie te verminderen? Waarom wel/niet? (Awareness)
3. Bent u op dit moment bereid om iets aan uw gedrag te veranderen om uw hersengezondheid te verbeteren en uw kans op dementie te verminderen? Waarom wel/niet? (Intention)

Deel 2: Specifiek gezondheidsgedrag

Op het registratieformulier hebben we u gevraagd naar uw gedrag. U heeft aangegeven dat u:

- **Rookt** [wel/niet]
- **Gemiddeld meer dan 1 glas alcohol per dag drinkt** [wel/niet]
- **Zichzelf niet als een actief persoon beschouwt** [wel/niet]
- **Niet gezond eet & niet mediterraan eet** [wel/niet]
- **Niet leergierig bent** [wel/niet] → bij niet stel ook vraag 4

Bespreek 1 gedrag: wel Roken, ≥1 glas alcohol p.d., niet actief, niet gezond eten

1. Waarom… (personal norms?)

- Rookt u?

- Drinkt u alcohol?

- Beschouwt u zichzelf niet als een actief persoon?

- Heeft u het gevoel dat u gezonder kunt eten?

Doorvragen:

1. Wat is het gezondheidsrisico van …? (Perceived threat, susceptibility)
2. Wat vindt u ervan dat … uw kans op dementie vergroot? (Knowledge, awareness, attitudes)
3. Zou u bereid zijn om … te veranderen om uw risico op dementie te verlagen?

   Wel → Waarom? En dan door naar vraag 2.

Niet → Direct door naar vraag 3

2. Stel u zou proberen om …. Wat zouden daar de voordelen van zijn?

(Attitude, belief)

- te stoppen met roken

- minder alcohol te drinken

- meer te bewegen

- gezonder te eten

Doorvragen:

1. Wat zijn de nadelen van …?
2. Wat heeft u nodig om …?
3. In welke situaties zou … lastig zijn? (Capacity, temptation)
4. In welke omgeving zou … moeilijk zijn? (Environmental influence)
5. In hoeverre hebben mensen die belangrijk voor u zijn invloed op uw keuze om …? (Social norm)
6. Wat zou u helpen om …? (Perceived behavioural control)

3. Waarom bent u niet bereid om …? (Willingness)

(Attitude, belief)

- te stoppen met roken

- minder alcohol te drinken

- meer te bewegen

- gezonder te eten

Doorvragen:

1. Wat zijn volgens u de voordelen van …? (Attitudes)
2. Welke rol speelt … in uw leven? (Habits)
3. Heeft u het gevoel dat u … als u het zou willen? (Perceived control). Waarom?
4. Wat zou er nodig zijn om u van gedachten te doen veranderen?

4. Op het registratieformulier heeft u aangegeven dat u zichzelf wel/niet als een leergierig persoon beschouwt. Waarom?

<u>Doorvragen:</u>

Onderzoek wijst uit dat leergierig zijn het belangrijkste gedrag is om uw hersengezondheid te bevorderen.

1. Wist u dit?

<u>Doorvragen niet leergierig:</u>

2. Ben u bereid om daar iets aan te veranderen? Waarom?
3. Wat zou u motiveren om er iets aan te veranderen?
4. Wat heeft u daarvoor nodig?

Afronding

We zijn inmiddels aan het eind van mijn vragenlijst beland. Is er iets dat u gemist heeft in dit gesprek?

Wilt u nog iets toevoegen aan hetgeen dat u vandaag verteld heeft?

Dan stel ik voor dat we het interview afronden. Ik wil u hartelijk bedanken voor uw deelname.

Study: Lifestyle and dementia

Introduction

In a life full of temptations and busy agendas it can be difficult to live healthy. With this study we investigate what motivates you to change your behavior in order to improve your lifestyle to improve your brain health and reduce the risk of dementia. During this 60-minute interview, I will ask you questions about your lifestyle. In the first part, we discuss what it actually means for you to live a healthy life and what reasons you have for changing your lifestyle or not. We are interested in your opinion, so you cannot give right or wrong answers.

In the second part of the interview, we discuss your answers on the registration form. More specifically, we discuss behaviors that are important for your brain health.

This interview is part of a scientific study. That is why I would like to discuss a number of preconditions with you before we start. We audio record this interview to carefully analyze the results. Therefore, I would like to switch on the audio recorder now and discuss your rights on tape.

A couple of days ago, you received an information letter. You already gave permission to participate in this study and the interview. I would like to emphasize that your participation is without any obligation. You may stop at any given time. Also during the interview. You do not have to provide a reason for quitting. If you tell us something during the conversation and would rather not have we use this in the analysis, please let us know. You can also do this after the interview. Our contact details are provided on the information letter you received.

1. We will record this interview using an audio recorder. **Do you think that's okay**?

2. In anonymous form we use a transcript of the interview to write a scientific report. This report is publicly accessible. **Do you object to that**?

3. We will make a transcript of this interview without the data that can be traced back to you. For example, we replace your name for a code. In order to enable other researchers to learn from our research, we make the transcribed interviews available on request. You have indicated on the consent form that you agree with this. **Can you confirm that on the audio-recording**?

Do you have any questions at this point? No? Then I suggest we start the interview.

Part 1: General perspectives on lifestyle

1. What do you perceive as a healthy lifestyle?

Sub question:

3. Do you have the idea you have a healthy lifestyle? Can you explain why?
4. What is the added value of a healthy lifestyle?
    a. What are long-term effects/benefits?
    b. What are short-term effects/benefits?

2. What can you do, at this moment, to live healthier?

Sub question:

2. You list a number of actions. What would be the most important one to you? Why?
    a. What if you would … What would you expect to happen? (Outcome expectancy)
    b. Ask sub questions about short-term and long-term effects.

3. Did you know that a healthy lifestyle can have benefits for your brain health and can reduce your future risk of dementia? (Knowledge)

Sub question:

Research shows that the risk of dementia is approximately 30-40% lower in people who do not smoke, exercise enough, drink little alcohol, eat a healthy diet, and are socially active and eager to learn.

4. How do you feel about this? (Attitude)
5. Do you think that you live healthy enough to reduce your personal risk for dementia? Could you elaborate on this? (Awareness)
6. Are you willing to change your behavior to improve your brain health and reduce your future risk for dementia? Could you explain your answer? (Intention)

Part 2: Specific behaviors

On the registration form we asked you about your behavior. On the form you indicated that you:

- **Smoke** [yes/no]
- **Consume more that 1 glass of alcohol per day** [yes/no]
- **Do not perceive yourself as a physically active person** [yes/no]
- **Do not perceive yourself as a person with a healthy / Mediterranean diet** [yes/no]
- **Are not eager to learn** [yes/no] → If the answer is no that also ask question 4

Discuss at least 1 behavior: smoking, high alcohol intake, physically inactive or unhealthy diet

1. Why do you [behavior]… (personal norm?)

- Smoke?

- Consume alcohol?

- Do not perceive yourself as a physically active person?

- Do not perceive yourself as a person with a healthy / Mediterranean diet?

Sub question:

4. What is the health risk/consequence of this behavior? (Perceived threat, susceptibility)
5. How do you feel about the fact this could increase your personal risk for dementia?

   (Knowledge, awareness, attitudes)
6. Would you be willing to change this behavior to reduce your personal risk for dementia?

   Yes → Why? Then continue with question 2.

   No → Directly continue with question 3.

2. Imagine you would try to … What would be a benefit?

(Attitude, belief)

- Quit smoking

- Consume less alcohol

- Perform more physical activity

- Adopt a healthier diet

Sub question:

7. What would be disadvantages?
8. What would you need to accomplish this?
9. In what situations would this be difficult to achieve? (Capacity, temptation)
10. In what environment would it be difficult to perform this behavior? (Environmental influence)
11. To what extent do people who are important to you influence your motivation to change this behavior? (Social norm)
12. What would help you? (Perceived behavioral control)

3. Why are you not willing to… (Willingness)

- Quit smoking
- Consume less alcohol
- Perform more physical activity
- Adopt a healthier diet

Sub question:

5. What would be advantages/disadvantages? (Attitudes)
6. What would be barriers?
7. What does this behavior mean to you? How is it part of your daily routine? (Habits)
8. Do you feel you would be able to change … if you would want this? Why? (Perceived control)
9. What is needed to change your mind in order to change this?

4. On the registration form you indicated that you consider yourself as a person who is not so eager to learn. Could you explain this?

Sub question:

Research shows that being eager to learn new things is an important behavior for brain health.

5. Did you know this?

<u>Sub question:</u>

6. Are you prepared/willing to change something about this? Why?
7. What would motivate you to change this?
8. What would you need?

End of the interview

We have now reached the end of the interview. Is there anything you missed during our conversation?

Would you like to add anything?

I would like to thank you very much for your participation.

Supplementary Table 2. Thematic data saturation

ID	Codes in document*	Fieldnotes	Quotes	Used codes*	Unique codes*	% Unique codes
1	149	0	1	81	81	100.0
3	141	0	1	89	66	74.2
10	91	2	1	65	34	52.3
17	95	1	3	78	40	51.3
24	77	2	2	54	23	42.6
12	116	2	1	68	16	23.5
21	113	2	1	70	36	51.4
2	73	1	2	51	14	27.5
15	102	0	1	59	20	33.9
22	108	1	1	78	19	24.4
28	78	2	0	59	12	20.3
30	86	3	1	66	21	31.8
35	128	2	3	79	13	16.5
39	116	1	2	83	10	12.0
13	81	0	0	54	11	20.4
9	74	0	3	54	2	3.7
25	93	3	3	71	4	5.6
29	97	0	3	75	3	4.0
8	90	0	1	63	1	1.6
19	113	0	2	77	5	6.5
26	118	4	5	70	4	5.7
14	92	1	3	62	2	3.2
32	74	0	0	59	2	3.4
**Total**		28	40	X	**467**	
* Excluded are fieldnotes en quotes						

Note. *The analyzed transcripts were randomly selected based on participant ID. This table presents the ID's in the order in which the transcripts were analyzed.*

Supplementary File 3. Slides used during member reflections (followed by an English Translation)

Vandaag

- Korte **introductie**
  - Terugblik op het onderzoek
- De **resultaten**
  - Daarover stel ik u een aantal vragen
  - Het gaat om uw mening (er is geen goed of fout)
- **Afronding en bedankje**
- Geschatte duur: **30 tot 40 minuten**

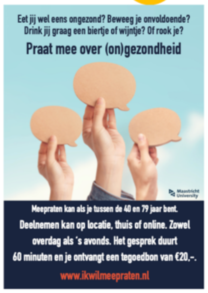

1

Introductie

- **Onderzoek over leefstijl en dementie**
- **Interviews** (*Januari-Maart 2022*)
  - 23 deelnemers tussen de 40 en 79 jaar
  - 15 vrouwen en 8 mannen
- **Opbouw van het interview**
  1. Gezonde leefstijl → Leeft u gezond? Wat kan er beter?
  2. Dementie risico → Ben u bereid uw levensstijl te veranderen?
  3. Veranderen van gedrag → Bewegen, eten, alcohol drinken, roken en/of leergierig zijn

2

De resultaten

3

Het risico op dementie

- **Positief over programma's die het risico op dementie verlagen**
  - Ervaring hebben met dementie (bijvoorbeeld via familie of werk)
- **Het risico op dementie niet duidelijk genoeg is**
  - "Hoeveel risico loop ik nu?"
  - "Dementie is nog ver in de toekomst."
- **Het effect van verandering van levensstijl is ook niet duidelijk**
  - "Hoeveel verlaag ik mijn risico als ik gedrag verander?" (bijvoorbeeld stop met roken)
  - "Succes is niet gegarandeerd."

4

Wisselende motivatie

- **Begin gesprek → Gemotiveerd om (algemeen) gezonder te leven**
  - Voordelen op korte termijn → "Ik zit lekker in mijn vel."
  - Voordelen op lange termijn → "Ik blijf langer zelfstandig."
- **Eind gesprek → Twijfels om (specifiek) gedrag te veranderen**
  - "Het is eigenlijk niet zo erg. Het valt wel mee."
  - "Ik compenseer met ander gezond gedrag."
  - "Ik heb de laatste jaren mijn levensstijl al verbeterd."
  - "Ik heb een wake-up call nodig."

5

Controle en zelfbeeld

- **Laag zelfbeeld → onvoldoende zelfzorg**
  - "Ik zet mezelf en mijn gezondheid en leefstijl op de tweede plek."
  - "Door omstandigheden kom ik niet toe aan een gezond leven."
- **Laag zelfbeeld → minder gevoel van controle**
  - "Ik ben lui, zwak" en "ik heb weinig wilskracht."
  - "Ik heb geen controle over mezelf (en mijn levensstijl)."

6

Today

- Short **introduction**
  - Recap on the study
- The **Results**
  - I will ask you some questions
  - Its about your perspective (no right or wrong)
- **Closing and thank you**
- Estimated time 30 to 40 minutes

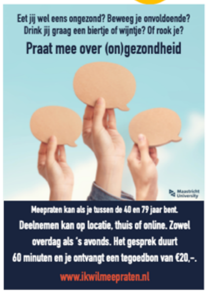

1

Introduction

- **Study about lifestyle and dementia**
- **Interviews** (*January-March 2022*)
  - 23 participants aged between 40 and 79 years
  - 15 female and 8 male
- **Structure of the interview**
  1. Healthy lifestyle → Do you life healthy? What can improve?
  2. Dementia risk → Are you open to change your lifestyle?
  3. Change behavior → Physical activity, diet, alcohol, smoking and cognitive activity

2

The results

3

Dementia risk

- **Positive about programs aimed at dementia risk reduction**
  - Experience with dementia (family or work)
- **Risk for dementia is not clear enough**
  - "what is my risk?"
  - "Dementia is still decades away."
- **Effect of lifestyle change is not clear enough**
  - "How much does my risk for dementia decreases if I change?" (e.g., stop smoking)
  - "No guarantee for success."

4

Alles of niets

- **Innerlijke strijd om leefstijl te veranderen**
  - "Ik moet de knop omzetten en ingrijpend veranderen."
    - "Ik ga geen chocolade meer eten." (at dagelijks chocolade)
    - "Ik moet meerdere keren per week hardlopen en meedoen met wedstijden." (sport niet)
- **Weinig realistische doelen**
  - Onduidelijkheid over richtlijnen → "Wat is gezond?" (hoeveel beweging?)
  - Behoefte aan coaching → "Alleen lukt het niet."

7

Afronding en bedankje

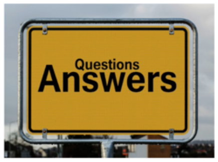

8

Ambivalent motivation

- **Start interview→ Motivated to have a (generic) healthier lifestyle**
  - Benefits short-term → "I feel good."
  - Benefits long-term → "I can stay independent."
- **End interview → Doubts about changing (specific) behavior**
  - "Its not that bad."
  - "I compensate with other health behavior."
  - "My lifestyle already improved during previous years."
  - "I need a wake-up call."

5

Controle and self-image

- **Low self-image → insufficient self-care**
  - "I put my health and lifestyle on the second place."
  - "Given barriers I am unable to life healthier."
- **Low self-image → less feeling of control**
  - "I am lazy, weak" and "I have no will-power."
  - "I have no control over myself (and my lifestyle)."

6

All or nothing

- **Inner struggle to change lifestyle**
  - "I need to flip a switch and rigorous change."
    - "I will not eat chocolate anymore." (eats chocolate daily)
    - "I need to go for a run multiple times a week and participate in running events." (doesn't exercise)
- **No realistic goals**
  - Unclear guidelines → "What is healthy?" (how many physical activity?)
  - Need for coaching→ "I can't do it alone."

7

Closing and thank you

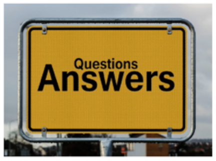

8

Supplementary File 4. Coding tree

Groups	Name	Groundedness	Creation Date
Alcohol - advice	drinking alcohol: advice from an internist	1	17-05-2022
Alcohol - advice	drinking alcohol: documentary	1	25-05-2022
Alcohol - advice	drinking alcohol: GP pointed out risks	1	25-07-2022

Groups	Name	Groundedness	Creation Date
Alcohol - change	alcohol drinken: self-efficacy	8	17-05-2022
Alcohol - change	drink alcohol: no alcohol at home	1	20-06-2022
Alcohol - change	drinking alcohol: awareness	5	17-06-2022
Alcohol - change	drinking alcohol: barriers when reducing use	4	10-05-2022
Alcohol - change	drinking alcohol: downsides of reducing use	3	10-05-2022
Alcohol - change	drinking alcohol: havent tried reducing	2	10-05-2022
Alcohol - change	drinking alcohol: having agreements	4	10-05-2022
Alcohol - change	drinking alcohol: motivation to reducing use	4	17-06-2022
Alcohol - change	drinking alcohol: not during the week	1	10-05-2022
Alcohol - change	drinking alcohol: reducing and awareness	2	10-05-2022
Alcohol - change	drinking alcohol: reducing due to sports	1	10-05-2022
Alcohol - change	drinking alcohol: reducing use	4	17-06-2022
Alcohol - change, H: Ambivilance	drinking alcohol: unwilling to reduce use	6	17-06-2022
Alcohol - change, H: Ambivilance	drinking alcohol: willing to reduce use	5	10-05-2022

Groups	Name	Groundedness	Creation Date
Alcohol - drinking, Demographics / medical background	addictions	6	02-05-2022
Alcohol - drinking	drinking alcohol: after divorce	1	21-06-2022
Alcohol - drinking	drinking alcohol: being drunk	1	12-05-2022
Alcohol - drinking	drinking alcohol: benefits	10	10-05-2022
Alcohol - drinking	drinking alcohol: biljarts	1	20-06-2022
Alcohol - drinking	drinking alcohol: burgundian lifestyle	1	17-05-2022
Alcohol - drinking	drinking alcohol: downside of use	5	10-05-2022
Alcohol - drinking	drinking alcohol: general	12	10-05-2022
Alcohol - drinking	drinking alcohol: habits	8	10-05-2022
Alcohol - drinking	drinking alcohol: health risks	4	17-05-2022
Alcohol - drinking	drinking alcohol: impact on sleep	1	18-07-2022
Alcohol - drinking	drinking alcohol: interaction with medication	1	17-05-2022
Alcohol - drinking	drinking alcohol: knowledge	1	17-06-2022
Alcohol - drinking	drinking alcohol: piece of mind	4	10-05-2022
Alcohol - drinking	drinking alcohol: problematic use	1	10-05-2022
Alcohol - drinking	drinking alcohol: relaxation	3	18-07-2022
Alcohol - drinking	drinking alcohol: reward	4	10-05-2022
Alcohol - drinking	drinking alcohol: risk	4	17-05-2022
Alcohol - drinking	drinking alcohol: social fun	5	17-06-2022
Alcohol - drinking	drinking alcohol: social norm	1	17-06-2022
Alcohol - drinking	drinking alcohol: social pressure	13	10-05-2022
Alcohol - drinking	drinking alcohol: tasty	5	17-05-2022

Alcohol - drinking	drinking alcohol: typical situations of use	1	20-06-2022
Alcohol - drinking	drinking alcohol: weekend	2	20-06-2022
Alcohol - drinking	drinking alcohol:daily	8	12-05-2022

Groups	Name	Groundedness	Creation Date
Behavior change	balance: cheat days	6	10-05-2022
Behavior change	balance: healthy vs unhealthy	8	10-05-2022
Behavior change, H: Ambivilance	behavior change is difficult	21	09-08-2022
Behavior change	behavior change: don't want to be pushed	2	08-08-2022
Behavior change, H: Ambivilance	behavior change: I have already changed	5	10-05-2022
Behavior change	behavior change: rewards	4	10-05-2022
Behavior change, Cognitive activity	cognitive activity: difficult to change	1	10-05-2022
Behavior change	motivation for behavior change: general	27	02-05-2022
Behavior change	willingness towards behavior change	4	26-08-2022
Behavior change	willingness towards behavior change for dementia risk reduction	8	09-08-2022

Groups	Name	Groundedness	Creation Date
Cognitive activity	cognitive activity: asking for additional explanation	1	25-07-2022
Cognitive activity	cognitive activity: be curious	2	17-05-2022
Cognitive activity	cognitive activity: become old aged	2	17-05-2022
Cognitive activity	cognitive activity: being cognitively slow	1	26-09-2022
Cognitive activity	cognitive activity: being interested in other people	1	17-05-2022
Cognitive activity,<br>H: Self-image	cognitive activity: being lazy	2	10-05-2022
Cognitive activity	cognitive activity: being socially involved	4	23-05-2022
Cognitive activity	cognitive activity: benefits	4	17-06-2022
Cognitive activity	cognitive activity: costs time/energy	1	10-08-2022
Cognitive activity	cognitive activity: critical thinking	1	10-05-2022
Cognitive activity	cognitive activity: definition	1	23-05-2022
Behavior change,<br>Cognitive activity	cognitive activity: difficult to change	1	10-05-2022
Cognitive activity	cognitive activity: doing things yourself	1	17-06-2022
Cognitive activity,<br>Dementia prevention - knowledge	cognitive activity: don't understand the link with dementia	2	20-06-2022
Cognitive activity	cognitive activity: finding new challenges	6	10-05-2022
Cognitive activity	cognitive activity: following a course	3	17-06-2022
Cognitive activity	cognitive activity: general	8	16-05-2022
Cognitive activity	cognitive activity: having an active lifestyle	4	17-05-2022
Cognitive activity	cognitive activity: increasing stimuli	1	17-06-2022

Cognitive activity	cognitive activity: keep working	1	03-06-2022
Cognitive activity	cognitive activity: lack of activities	1	20-06-2022
Cognitive activity	cognitive activity: learn a new language	1	17-06-2022
Cognitive activity	cognitive activity: look for background information	5	10-05-2022
Cognitive activity	cognitive activity: mind games	4	12-05-2022
Cognitive activity	cognitive activity: no need to change	2	23-05-2022
Cognitive activity	cognitive activity: not reading	2	26-08-2022
Cognitive activity	cognitive activity: playing games	2	17-06-2022
Cognitive activity	cognitive activity: playing music	2	18-08-2022
Cognitive activity	cognitive activity: profession	2	25-05-2022
Cognitive activity	cognitive activity: reading	5	12-05-2022
Cognitive activity	cognitive activity: relation to career ambitions	3	17-05-2022
Cognitive activity	cognitive activity: societal involvement	4	26-08-2022
Cognitive activity	cognitive activity: stress	2	17-05-2022
Cognitive activity	cognitive activity: trigger your brains	4	17-05-2022
Cognitive activity	cognitive activity: using soical media	1	10-08-2022
Cognitive activity	cognitive activity:following the news	4	17-05-2022

Groups	Name	Groundedness	Creation Date
Covid-19	covid-19: diet	2	18-08-2022
Covid-19	covid-19: gaining weight	1	12-11-2022
Covid-19	covid-19: physical activity	14	25-05-2022
Covid-19	covid-19: skeptical	2	20-06-2022
Covid-19	covid-19: stress	4	10-05-2022
Covid-19	effects of covid-19 pandemic	1	02-05-2022

Groups	Name	Groundedness	Creation Date
Dementia prevention,<br>H: Dementia prevention - abstract	dementia is horrible	3	17-06-2022
Dementia prevention,<br>H: Dementia prevention - abstract	dementia risk reduction: own interpretation	26	25-05-2022
Dementia prevention	drinking alcohol: dementia risk	6	10-05-2022
Dementia prevention	healthy lifestyle and dementia	1	02-05-2022
Dementia prevention	neuropsychological assessment: concussion	1	20-06-2022

Groups	Name	Groundedness	Creation Date
Cognitive activity,<br>Dementia prevention - knowledge	cognitive activity: don't understand the link with dementia	2	20-06-2022
Dementia prevention - knowledge,<br>H: Dementia prevention - abstract	dementia risk reduction: genetic influence	3	17-06-2022
Dementia prevention - knowledge,<br>H: Dementia prevention - abstract	dementia risk reduction: knowledge and awareness	48	02-05-2022

Groups	Name	Groundedness	Creation Date
Dementia prevention - openness,<br>H: Ambivilance,<br>H: Dementia prevention - abstract	dementia risk reduction: motivation	33	02-05-2022
Dementia prevention - openness,<br>H: Dementia prevention - abstract	dementia risk reduction: perceptions	13	12-05-2022
Dementia prevention - openness,<br>H: Dementia prevention - abstract	dementia risk reduction: willingness	7	10-05-2022
Dementia prevention - openness,<br>H: Dementia prevention - abstract	dementia risk reduction: worries about memory loss	6	12-05-2022
Dementia prevention - openness,<br>Demographics / medical background,<br>H: Dementia prevention - abstract	experience with dementia: family or work	18	25-05-2022

Groups	Name	Groundedness	Creation Date
Alcohol - drinking, Demographics / medical background	addictions	6	02-05-2022
Demographics / medical background, Physical effects	artrosis	2	26-08-2022
Demographics / medical background	children	2	02-05-2022
Demographics / medical background, Other	domestic violence	1	30-05-2022
Dementia prevention - openness, Demographics / medical background, H: Dementia prevention - abstract	experience with dementia: family or work	18	25-05-2022
Demographics / medical background	family history with cardiovascular disease	4	25-05-2022
Demographics / medical background	family history with lung disease	2	25-07-2022
Demographics / medical background, Overweight	family history with obesity	1	08-08-2022
Demographics / medical background, Well being	homeless	1	20-06-2022
Demographics / medical background	LHBTI	1	09-08-2022
Demographics / medical background, H: Ambivilance	lifestyle: unhealthy history	9	02-05-2022
Demographics / medical background	personality	8	17-06-2022
Demographics / medical background	profession: employment and education	13	23-05-2022
Demographics / medical background	retirement	6	15-09-2022

Groups	Name	Groundedness	Creation Date
Diet	healthy diet: risks of unhealthy diet	6	10-05-2022
Diet	healthy diet: benefits	4	30-05-2022
Diet	healthy diet: butter	1	02-09-2022
Diet	healthy diet: difficult when you eat outdoors	1	04-07-2022
Diet	healthy diet: does not taste good	3	12-05-2022
Diet	healthy diet: doing groceries on a daily basis	1	17-06-2022
Diet	healthy diet: don't take any treats	2	15-08-2022
Diet	healthy diet: eating and TV	1	17-06-2022
Diet	healthy diet: eating to little	3	20-06-2022
Diet	healthy diet: effect is increased energy	9	10-05-2022
Diet	healthy diet: effect is more stamina	3	10-05-2022
Diet	healthy diet: effect on physical health	11	31-05-2022
Diet	healthy diet: enjoying cooking	1	17-06-2022
Diet	healthy diet: environmental influences	7	17-06-2022
Diet	healthy diet: exhausted at the end of my day	2	17-06-2022
Diet	healthy diet: fasting	1	25-05-2022
Diet,<br>H: Self-image	healthy diet: feeling good about yourself	1	17-06-2022
Diet	healthy diet: financial costs	3	12-05-2022
Diet	healthy diet: finding a balance	2	17-06-2022
Diet	healthy diet: following a diet	4	01-06-2022
Diet,<br>H: Self-image	healthy diet: fulfilment	3	18-08-2022

Diet	healthy diet: gaining weight	4	12-05-2022
Diet	healthy diet: general	6	10-05-2022
Diet	healthy diet: habits	6	12-05-2022
Diet,<br>H: Goals	healthy diet: having (no) routine	4	17-06-2022
Diet	healthy diet: having avaried diet	6	10-08-2022
Diet	healthy diet: inhibition problems	3	26-09-2022
Diet	healthy diet: leads to discussion with family	3	10-05-2022
Diet	healthy diet: less salt	5	17-06-2022
Diet	healthy diet: living alone	10	31-05-2022
Diet	healthy diet: monotonous	1	12-05-2022
Diet	healthy diet: no breakfast	1	20-06-2022
Diet,<br>H: Dementia prevention - abstract	healthy diet: no clear link with dementia	1	17-05-2022
Diet,<br>H: Goals	healthy diet: no goal	1	17-06-2022
Diet	healthy diet: not enjoying cooking	2	17-06-2022
Diet	healthy diet: obesity	7	01-06-2022
Diet	healthy diet: patterns	1	20-06-2022
Diet	healthy diet: perceptions about own diet	14	16-05-2022
Diet	healthy diet: responsibility of politics/industry	7	12-05-2022
Diet,<br>H: Self-image	healthy diet: self-efficacy	7	12-05-2022
Diet	healthy diet: social influences	25	10-05-2022
Diet	healthy diet: social norm	5	04-07-2022
Diet	healthy diet: sugar addiction	6	17-06-2022
Diet	healthy diet: suggar tax	1	17-06-2022

Diet	healthy diet: supplements	5	25-05-2022
Diet	healthy diet: the convenience of unhealthy food	4	12-05-2022
Diet	healthy diet: time consuming	5	17-06-2022
Diet	healthy diet: unhealthy foods	2	12-07-2022
Diet	healthy diet: weighing healthy vs unhealthy choices	5	16-05-2022
Diet	healthy diet: works better at home	1	17-06-2022
Diet	unhealthy diet: but I gain no weigth	1	17-06-2022
Diet	unhealthy diet: general	5	18-08-2022
Diet,<br>H: Self-image	unhealthy diet: guilt	4	18-08-2022
Diet	unhealthy diet: influence of work	7	17-06-2022
Diet	unhealthy diet: lack of structure during the day	2	11-07-2022
Diet	unhealthy diet: snacks	11	03-06-2022
Diet	unhealthy diet: temptations in the supermarket	1	03-06-2022

Groups	Name	Groundedness	Creation Date
Diet - change	healthy diet: awareness	12	12-05-2022
Diet - change	healthy diet: bariers	5	16-05-2022
Diet - change	healthy diet: behavior change	3	17-06-2022
Diet - change, H: Ambivilance	healthy diet: compensating	8	16-05-2022
Diet - change	healthy diet: creating new routines	3	10-05-2022
Diet - change	healthy diet: guided practice	2	17-06-2022
Diet - change	healthy diet: having a plan	2	17-06-2022
Diet - change, H: Goals	healthy diet: I know it but don't do it	2	10-05-2022
Diet - change	healthy diet: is difficult	10	12-07-2022
Diet - change	healthy diet: no inspiration to eat healthy	1	11-07-2022
Diet - change	healthy diet: reading food labels	3	12-05-2022
Diet - change	healthy diet: set priorities	4	17-06-2022

Groups	Name	Groundedness	Creation Date
Fieldnotes,<br>H: Ambivilance	fieldnote: again, a participants mentions halfway the interview to smoke	2	01-10-2022
Fieldnotes,<br>H: Dementia prevention - abstract	fieldnote: again, there needs to be a specific reward/benefit	1	06-11-2022
Fieldnotes	fieldnote: already participates in different research	1	18-07-2022
Fieldnotes,<br>H: Ambivilance	fieldnote: ambivalence about heatlhy lifestyle behavior	7	24-05-2022
Fieldnotes	fieldnote: compared to smoking this leads to more self-efficacy	1	26-08-2022
Fieldnotes,<br>H: Ambivilance	fieldnote: contradictory	1	17-05-2022
Fieldnotes,<br>H: Ambivilance	fieldnote: downplays effects of alcohol use	5	25-05-2022
Fieldnotes,<br>H: Ambivilance	fieldnote: downplays the severity	1	26-08-2022
Fieldnotes,<br>H: Ambivilance	fieldnote: earlier conversation seems to point out something else; eats unhealthy and does not participate in sports	1	09-08-2022
Fieldnotes,<br>H: Dementia prevention - abstract	fieldnote: good quote to illustrate the bottle neck of prevention	1	08-08-2022
Fieldnotes,<br>H: Goals	fieldnote: has no specific plans to change behavior	1	31-05-2022
Fieldnotes,<br>H: Ambivilance	fieldnote: have the idea she is not willing to talk about this	2	17-06-2022
Fieldnotes	fieldnote: illustration of a main theme?	1	01-10-2022

Fieldnotes,<br>H: Ambivilance	fieldnote: initially expresses willingness to quit smoking but now stresses to need a wake-up call. Later tells about a cardiovascular event.	1	03-06-2022
Fieldnotes	fieldnote: is supprises that alcohol use is not discussed (only low consumption)	1	15-09-2022
Fieldnotes	fieldnote: lives together with his brother	1	20-06-2022
Fieldnotes	Fieldnote: maybe we should separate smoking from wake-up call?	1	03-06-2022
Fieldnotes	fieldnote: medicalizes behavior change	1	15-09-2022
Fieldnotes	fieldnote: seems confronting to talk about unhealthy behaviors	1	05-10-2022
Fieldnotes	fieldnote: seems more motivated after the interview.	1	25-07-2022
Fieldnotes,<br>H: Ambivilance	fieldnote: skeptical	1	18-08-2022
Fieldnotes	fieldnote: sums up many health risks	1	20-06-2022
Fieldnotes	fieldnote: talks a lot about what she knows about science but not so much about her own behavior	1	24-05-2022
Fieldnotes	fieldnote: unclear guidelines	4	17-05-2022
Fieldnotes	fieldnote: underreported alcohol use on the registration form	1	26-09-2022
Fieldnotes	fielnote: went to a recovery clinic	1	20-06-2022
Fieldnotes	quote	40	10-05-2022

Groups	Name	Groundedness	Creation Date
Behavior change,<br>H: Ambivilance	behavior change is difficult	21	09-08-2022
Behavior change,<br>H: Ambivilance	behavior change: I have already changed	5	10-05-2022
Dementia prevention - openness,<br>H: Ambivilance,<br>H: Dementia prevention - abstract	dementia risk reduction: motivation	33	02-05-2022
Alcohol - change,<br>H: Ambivilance	drinking alcohol: unwilling to reduce use	6	17-06-2022
Alcohol - change,<br>H: Ambivilance	drinking alcohol: willing to reduce use	5	10-05-2022
Fieldnotes,<br>H: Ambivilance	fieldnote: again, a participants mentions halfway the interview to smoke	2	01-10-2022
Fieldnotes,<br>H: Ambivilance	fieldnote: ambivalence about heatlhy lifestyle behavior	7	24-05-2022
Fieldnotes,<br>H: Ambivilance	fieldnote: contradictory	1	17-05-2022
Fieldnotes,<br>H: Ambivilance	fieldnote: downplays effects of alcohol use	5	25-05-2022
Fieldnotes,<br>H: Ambivilance	fieldnote: downplays the severity	1	26-08-2022
Fieldnotes,<br>H: Ambivilance	fieldnote: earlier conversation seems to point out something else; eats unhealthy and does not participate in sports	1	09-08-2022
Fieldnotes,<br>H: Ambivilance	fieldnote: have the idea she is not willing to talk about this	2	17-06-2022
Fieldnotes,<br>H: Ambivilance	fieldnote: initially expresses willingness to quit smoking but now stresses to need a wake-up call. Later tells about a cardiovascular event.	1	03-06-2022
Fieldnotes,<br>H: Ambivilance	fieldnote: skeptical	1	18-08-2022

Diet - change,<br>H: Ambivilance	healthy diet: compensating	8	16-05-2022
H: Ambivilance	in need for a wake-up call	10	03-06-2022
Demographics / medical background,<br>H: Ambivilance	lifestyle: unhealthy history	9	02-05-2022
H: Ambivilance, Lifestyle - general	own perception about healthy living	22	12-07-2022
H: Ambivilance,<br>Physical activity - change	physical activity: internal struggle	13	25-05-2022
H: Ambivilance,<br>Lifestyle - general	unwilling to live healthy	4	17-06-2022

Groups	Name	Groundedness	Creation Date
Dementia prevention,<br>H: Dementia prevention - abstract	dementia is horrible	3	17-06-2022
Dementia prevention - knowledge,<br>H: Dementia prevention - abstract	dementia risk reduction: genetic influence	3	17-06-2022
Dementia prevention - knowledge,<br>H: Dementia prevention - abstract	dementia risk reduction: knowledge and awareness	48	02-05-2022
Dementia prevention - openness,<br>H: Ambivilance,<br>H: Dementia prevention - abstract	dementia risk reduction: motivation	33	02-05-2022
Dementia prevention,<br>H: Dementia prevention - abstract	dementia risk reduction: own interpretation	26	25-05-2022
Dementia prevention - openness,<br>H: Dementia prevention - abstract	dementia risk reduction: perceptions	13	12-05-2022
Dementia prevention - openness,<br>H: Dementia prevention - abstract	dementia risk reduction: willingness	7	10-05-2022
Dementia prevention - openness,<br>H: Dementia prevention - abstract	dementia risk reduction: worries about memory loss	6	12-05-2022
Dementia prevention - openness,<br>Demographics / medical background,<br>H: Dementia prevention - abstract	experience with dementia: family or work	18	25-05-2022
Fieldnotes,<br>H: Dementia prevention - abstract	fieldnote: again, there needs to be a specific reward/benefit	1	06-11-2022
Fieldnotes,<br>H: Dementia prevention - abstract	fieldnote: good quote to illustrate the bottle neck of prevention	1	08-08-2022
Diet,<br>H: Dementia prevention - abstract	healthy diet: no clear link with dementia	1	17-05-2022
H: Dementia prevention - abstract,<br>Physical activity - change	physical activity: skeptical about benefits	2	06-11-2022

Groups	Name	Groundedness
H: Goals,<br>Physical activity - change	being overly ambitious	8
Fieldnotes,<br>H: Goals	fieldnote: has no specific plans to change behavior	1
Diet,<br>H: Goals	healthy diet: having (no) routine	4
Diet - change,<br>H: Goals	healthy diet: I know it but don't do it	2
Diet,<br>H: Goals	healthy diet: no goal	1
H: Goals,<br>Tools for behavior change	social life: action planning	3
H: Goals,<br>Tools for behavior change	tools: goal setting	8

Creation Date

03-10-2022

31-05-2022

17-06-2022

10-05-2022

17-06-2022

09-08-2022

25-05-2022

Groups	Name	Groundedness	Creation Date
Cognitive activity, H: Self-image	cognitive activity: being lazy	2	10-05-2022
H: Self-image	diet: limited self-care	6	30-05-2022
Diet, H: Self-image	healthy diet: feeling good about yourself	1	17-06-2022
Diet, H: Self-image	healthy diet: fulfilment	3	18-08-2022
Diet, H: Self-image	healthy diet: self-efficacy	7	12-05-2022
H: Self-image, Lifestyle - general	healthy lifestyle: being proud about yourself	2	17-06-2022
H: Self-image, Lifestyle - general	healthy lifestyle: feeling good	15	12-05-2022
H: Self-image	healthy lifestyle: gives self-confidence	4	11-07-2022
H: Self-image	healthy lifestyle: limited self-control	1	17-05-2022
H: Self-image, Lifestyle - general	healthy lifestyle: self-efficacy	3	20-06-2022
H: Self-image	limited self-care	9	05-09-2022
H: Self-image	negative self-image	12	10-08-2022
H: Self-image, Physical activity	physical activity: feeling good about yourself	2	17-06-2022
H: Self-image, Physical activity	physical activity: gaining self-confidence	4	12-05-2022
H: Self-image, Physical activity	physical activity: lazy	7	25-05-2022
H: Self-image, Tools for behavior change	tool: working on self-image	1	31-10-2022
Diet, H: Self-image	unhealthy diet: guilt	4	18-08-2022

Groups	Name	Groundedness	Creation Date
Lifestyle - general	healthy lifestyle: (grand)children	3	17-06-2022
Lifestyle - general	healthy lifestyle: becoming old	21	17-05-2022
Lifestyle - general	healthy lifestyle: being a caregiver	1	17-06-2022
Lifestyle - general	healthy lifestyle: being able to do something for someone else	1	12-05-2022
Lifestyle - general	healthy lifestyle: being outdoors	6	12-05-2022
Lifestyle - general	healthy lifestyle: being positive	1	12-05-2022
H: Self-image, Lifestyle - general	healthy lifestyle: being proud about yourself	2	17-06-2022
Lifestyle - general	healthy lifestyle: cardiovascular health	5	03-06-2022
Lifestyle - general	healthy lifestyle: current lifestyle	7	02-05-2022
Lifestyle - general	healthy lifestyle: divorce	1	20-06-2022
Lifestyle - general	healthy lifestyle: doesn't care about the future	4	17-06-2022
Lifestyle - general	healthy lifestyle: downside of a healthy lifestyle	4	02-05-2022
Lifestyle - general	healthy lifestyle: effect	26	02-05-2022
Lifestyle - general	healthy lifestyle: effect age	7	02-05-2022
Lifestyle - general	healthy lifestyle: effect of unhealthy behavior	6	02-05-2022
Lifestyle - general	healthy lifestyle: environmental influences	1	02-05-2022
Lifestyle - general	healthy lifestyle: fait	2	12-05-2022
H: Self-image, Lifestyle - general	healthy lifestyle: feeling good	15	12-05-2022
Lifestyle - general	healthy lifestyle: health benefits	2	10-05-2022
Lifestyle - general	healthy lifestyle: infleunce of cosmetics	2	12-05-2022
Lifestyle - general	healthy lifestyle: influence of life-events	3	03-06-2022

Lifestyle - general	healthy lifestyle: influence of plastic	1	12-05-2022
Lifestyle - general	healthy lifestyle: living conditions	1	01-10-2022
Lifestyle - general	healthy lifestyle: long-term effect	23	02-05-2022
Lifestyle - general	healthy lifestyle: mid-life	3	17-05-2022
Lifestyle - general	healthy lifestyle: obesity	7	16-05-2022
Lifestyle - general	healthy lifestyle: own definition	32	02-05-2022
Lifestyle - general	healthy lifestyle: own perception	21	02-05-2022
Lifestyle - general	healthy lifestyle: own responsibility	2	27-10-2022
Lifestyle - general	healthy lifestyle: parental education	3	10-08-2022
Lifestyle - general	healthy lifestyle: personality	4	17-06-2022
Lifestyle - general	healthy lifestyle: room for improvement	23	02-05-2022
H: Self-image, Lifestyle - general	healthy lifestyle: self-efficacy	3	20-06-2022
Lifestyle - general	healthy lifestyle: short-term effect	22	02-05-2022
Lifestyle - general	healthy lifestyle: sleep patterns	10	10-05-2022
Lifestyle - general	healthy lifestyle: social environment	10	02-05-2022
Lifestyle - general	healthy lifestyle: social influence	6	12-05-2022
Lifestyle - general	healthy lifestyle: spiritual	2	16-05-2022
Lifestyle - general	healthy lifestyle: stamina	8	20-06-2022
Lifestyle - general	healthy lifestyle: stay young	5	10-05-2022
Lifestyle - general	healthy lifestyle: unclear guidelines	9	17-05-2022
Lifestyle - general	healthy lifestyle: vision about health	3	16-05-2022
Lifestyle - general	healthy lifestyle: worries about unhealthy lifestyle	9	02-05-2022

Lifestyle - general, Medical history	medical history: medical conditions	4	17-06-2022
Lifestyle - general, Medical history	medical history: medication	8	12-05-2022
H: Ambivilance, Lifestyle - general	own perception about healthy living	22	12-07-2022
H: Ambivilance, Lifestyle - general	unwilling to live healthy	4	17-06-2022

Groups	Name	Groundedness	Creation Date
Lifestyle - general, Medical history	medical history: medical conditions	4	17-06-2022
Lifestyle - general, Medical history	medical history: medication	8	12-05-2022
Medical history	medical history: psychological problems	1	11-07-2022

Groups	Name	Groundedness	Creation Date
Other	care needs	1	02-10-2022
Demographics / medical background, Other	domestic violence	1	30-05-2022
Other	hobbies	1	02-05-2022
Other, Tools for behavior change	tools: sports for elderly	2	17-06-2022

Groups	Name	Groundedness	Creation Date
Demographics / medical background, Overweight	family history with obesity	1	08-08-2022
Overweight	obesity: benefits of losing weight	3	03-06-2022
Overweight	obesity: confronting	4	18-08-2022
Overweight	obesity: diet	2	03-06-2022
Overweight	obesity: jojo-effect	1	03-06-2022
Overweight	obesity: shame	1	23-08-2022
Overweight	obesity: societal perspective	3	18-08-2022

Groups	Name	Groundedness	Creation Date
Physical activity	physical activity: routine	4	10-08-2022
Physical activity	physical activity: (un)convinience	6	20-06-2022
Physical activity	physical activity: being an active person	6	08-08-2022
Physical activity	physical activity: being outdoors	3	12-05-2022
Physical activity	physical activity: benefits	17	16-05-2022
Physical activity	physical activity: bike	12	13-06-2022
Physical activity	physical activity: difficulty	6	25-05-2022
Physical activity	physical activity: discipline	1	18-08-2022
Physical activity	physical activity: dislike sports	11	12-05-2022
Physical activity	physical activity: exhaustion	3	12-05-2022
Physical activity	physical activity: feeling good	9	10-05-2022
H: Self-image, Physical activity	physical activity: feeling good about yourself	2	17-06-2022
Physical activity	physical activity: feels unconfortable	1	09-09-2022
Physical activity	physical activity: financial burden	1	12-05-2022
H: Self-image, Physical activity	physical activity: gaining self-confidence	4	12-05-2022
Physical activity	physical activity: gardening	1	17-06-2022
Physical activity	physical activity: general	8	10-05-2022
Physical activity	physical activity: gym	8	08-08-2022
Physical activity	physical activity: health risks	9	16-05-2022
Physical activity	physical activity: improved breathing	4	12-05-2022
H: Self-image, Physical activity	physical activity: lazy	7	25-05-2022
Physical activity	physical activity: long-term effect	9	16-05-2022

Physical activity	physical activity: more energy	10	12-05-2022
Physical activity	physical activity: more strength	2	15-08-2022
Physical activity	physical activity: motivates to live heatlhier	3	10-05-2022
Physical activity	physical activity: negative effects on not enough	2	12-05-2022
Physical activity	physical activity: negative influence werk/education	12	24-05-2022
Physical activity	physical activity: not enough	17	16-05-2022
Physical activity	physical activity: obesity	6	10-08-2022
Physical activity	physical activity: profession	5	17-06-2022
Physical activity	physical activity: relaxation	2	09-08-2022
Physical activity	physical activity: scooter	3	12-05-2022
Physical activity	physical activity: sedentary behavior	10	12-05-2022
Physical activity	physical activity: seizonal effects	12	23-05-2022
Physical activity	physical activity: self-satisfaction	3	25-05-2022
Physical activity	physical activity: social comparison	7	12-05-2022
Physical activity	physical activity: social influence	27	12-05-2022
Physical activity	physical activity: sports	12	10-05-2022
Physical activity	physical activity: taking it easy	2	21-06-2022
Physical activity	physical activity: taking the car	4	08-08-2022
Physical activity	physical activity: the environment	4	12-05-2022
Physical activity	physical activity: time restraints	13	25-05-2022

Physical activity	physical activity: walking	16	17-06-2022
Physical activity	physical activity: well-being	3	12-05-2022

Groups	Name	Groundedness	Creation Date
Physical activity - advice	physical activity: knowlegde on guidelines	7	18-08-2022

Groups	Name	Groundedness	Creation Date
H: Goals,<br>Physical activity - change	being overly ambitious	8	03-10-2022
Physical activity - change	physical activity: barriers	31	16-05-2022
Physical activity - change	physical activity: breaking with routines	6	15-08-2022
Physical activity - change	physical activity: downsides	7	12-05-2022
Physical activity - change	physical activity: goal attainment	10	10-05-2022
Physical activity - change	physical activity: goal setting	5	10-05-2022
Physical activity - change	physical activity: having a dog	10	12-05-2022
H: Ambivilance,<br>Physical activity - change	physical activity: internal struggle	13	25-05-2022
Physical activity - change	physical activity: no need for change	1	06-11-2022
Physical activity - change	physical activity: rules	2	16-05-2022
Physical activity - change	physical activity: self-reflection	2	25-05-2022
H: Dementia prevention - abstract,<br>Physical activity - change	physical activity: skeptical about benefits	2	06-11-2022
Physical activity - change	physical activity: there is always room for improvement	2	26-09-2022

Groups	Name	Groundedness	Creation Date
Demographics / medical background, Physical effects	artrosis	2	26-08-2022
Physical effects	breathing	4	10-08-2022
Physical effects	cholesterol	2	02-09-2022
Physical effects	COPD: physcial complaints	3	20-06-2022
Physical effects	hearing problems	1	02-09-2022
Physical effects	physical activity: physical complaints	25	30-05-2022
Physical effects	worries about high blood pressure	4	09-09-2022

Groups	Name	Groundedness	Creation Date
Prevention	knowledge lifestyle in general	15	23-05-2022

Groups	Name	Groundedness	Creation Date
Sleep	sleep: negative effects bad sleep	1	27-10-2022
Sleep, Well being	sleep: stress	2	27-10-2022
Sleep	sleep: turning aroung day-night	3	27-10-2022

Groups	Name	Groundedness	Creation Date
Smoking	smoking: addiction	6	17-06-2022
Smoking	smoking: alcohol	6	09-08-2022
Smoking	smoking: barriers to quit	6	30-05-2022
Smoking	smoking: cancer	1	26-09-2022
Smoking	smoking: COPD	3	31-05-2022
Smoking	smoking: dementia risk	1	30-05-2022
Smoking	smoking: emotional situation	1	01-10-2022
Smoking	smoking: environment	11	30-05-2022
Smoking	smoking: expensive	3	03-06-2022
Smoking	smoking: family	1	15-09-2022
Smoking	smoking: general	3	10-05-2022
Smoking	smoking: healthy risk	4	17-06-2022
Smoking	smoking: more on days off	1	15-09-2022
Smoking	smoking: more physical activity	1	31-05-2022
Smoking	smoking: negative aspects	5	30-05-2022
Smoking	smoking: no	3	16-05-2022
Smoking	smoking: not indoors	2	31-05-2022
Smoking	smoking: obesity	4	17-06-2022
Smoking	smoking: pregnancy	1	31-05-2022
Smoking	smoking: relaxation	5	30-05-2022
Smoking	smoking: responsibility politics/industry	1	17-06-2022
Smoking	smoking: reward	2	30-05-2022
Smoking	smoking: saving money	4	31-05-2022
Smoking	smoking: seeking distraction	1	15-09-2022
Smoking	smoking: social influence	15	10-05-2022
Smoking	smoking: societal perspective	1	15-09-2022
Smoking	smoking: stamina	1	09-08-2022
Smoking	smoking: stress	8	31-05-2022
Smoking	smoking: taste	2	09-08-2022
Smoking	smoking: worries about health	2	03-06-2022
Smoking	smoking: yes	3	30-05-2022

Groups	Name	Groundedness	Creation Date
Smoking - change	smoking: attempts to quit	9	08-08-2022
Smoking - change	smoking: benefits of quiting	11	30-05-2022
Smoking - change	smoking: compensating with food	5	03-06-2022
Smoking - change	smoking: difficulty of quiting	8	30-05-2022
Smoking - change	smoking: habit	13	03-06-2022
Smoking - change	smoking: motivation to quit	14	30-05-2022
Smoking - change	smoking: negative aspects of quiting	7	30-05-2022
Smoking - change	smoking: professional help	5	31-05-2022
Smoking - change	smoking: quiting makes you aware	1	09-08-2022
Smoking - change	smoking: reduction	3	31-05-2022
Smoking - change	smoking: relapse	6	09-08-2022
Smoking - change	smoking: self-efficacy	11	31-05-2022
Smoking - change	smoking: stopped	6	11-07-2022
Smoking - change	smoking: tools that help you stop	11	30-05-2022

Groups	Name	Groundedness	Creation Date
Social life	social life:	3	02-09-2022
Social life	social life: being a loner	9	12-05-2022
Social life	social life: general	2	17-06-2022
Social life	social life: grandchildren	1	17-06-2022
Social life	social life: hearing problems	2	02-09-2022
Social life	social life: showing initiative	2	02-09-2022
Social life	social life: work	1	17-06-2022

Groups	Name	Groundedness	Creation Date
H: Goals,<br>Tools for behavior change	social life: action planning	3	09-08-2022
Tools for behavior change	social life: advice physician	1	26-08-2022
H: Self-image,<br>Tools for behavior change	tool: working on self-image	1	31-10-2022
Tools for behavior change	tools: a bike plan	1	10-08-2022
Tools for behavior change	tools: a book	1	26-09-2022
Tools for behavior change	tools: a standup desk	1	24-05-2022
Tools for behavior change	tools: a tool to count steps	3	10-05-2022
Tools for behavior change	tools: agreements	2	16-05-2022
Tools for behavior change	tools: app	6	18-08-2022
Tools for behavior change	tools: avoiding triggers	2	08-08-2022
Tools for behavior change	tools: awareness	8	10-05-2022
Tools for behavior change	tools: balance days	3	12-05-2022
Tools for behavior change	tools: campagne	4	17-06-2022
Tools for behavior change	tools: cardiofitness	1	03-06-2022
Tools for behavior change	tools: changing old habits	3	17-06-2022
Tools for behavior change	tools: changing your mindset	3	23-08-2022
Tools for behavior change	tools: coaching	21	24-05-2022
Tools for behavior change	tools: drinking water	2	17-06-2022
Tools for behavior change	tools: games to excersise	1	24-05-2022
H: Goals,<br>Tools for behavior change	tools: goal setting	8	25-05-2022
Tools for behavior change	tools: going with retirement	1	12-11-2022
Tools for behavior change	tools: guided practice	2	17-06-2022
Tools for behavior change	tools: healthy food	4	16-05-2022
Tools for behavior change	tools: home fitness program	2	24-05-2022
Tools for behavior change	tools: information	3	17-06-2022
Tools for behavior change	tools: Iwatch	3	24-05-2022
Tools for behavior change	tools: living healthy	1	02-05-2022
Tools for behavior change	tools: mindfulness	2	03-10-2022
Tools for behavior change	tools: new routines	11	10-05-2022
Tools for behavior change	tools: no smoking with friends	1	17-06-2022

Tools for behavior change	tools: not smoking in the car	1	17-06-2022
Tools for behavior change	tools: nutritionist	1	17-06-2022
Tools for behavior change	tools: ordering a meal box	2	10-08-2022
Tools for behavior change	tools: physical activity	2	16-05-2022
Tools for behavior change	tools: prescription	1	10-05-2022
Tools for behavior change	tools: receiving feedback	1	27-10-2022
Tools for behavior change	tools: rewards	4	10-05-2022
Tools for behavior change	tools: self-reflection	5	12-05-2022
Tools for behavior change	tools: self-relaxation exercise	4	23-05-2022
Tools for behavior change	tools: should not be obligated	1	09-08-2022
Tools for behavior change	tools: social groups	2	09-08-2022
Tools for behavior change	tools: social pressure	8	25-05-2022
Other,<br>Tools for behavior change	tools: sports for elderly	2	17-06-2022
Tools for behavior change	tools: support from peers	2	08-08-2022
Tools for behavior change	tools: tips	1	10-08-2022
Tools for behavior change	tools: treadmill	2	12-05-2022

Groups	Name	Groundedness	Creation Date
Well being	balance: physical - mental health	12	11-07-2022
Well being	balnce: importance	8	08-08-2022
Well being	healthy lifestyle: increased well-being	13	25-05-2022
Demographics / medical background, Well being	homeless	1	20-06-2022
Well being	Living right now	4	15-09-2022
Well being	Mental well-being	8	16-05-2022
Well being	Mental well-being: being outdoors	1	17-06-2022
Well being	Mental well-being: depression	2	31-05-2022
Well being	Mental well-being: divorce	1	20-06-2022
Well being	Mental well-being: having a pet	1	17-06-2022
Well being	Mental well-being: physical activity	1	17-06-2022
Well being	Mental well-being: social contacts	3	17-06-2022
Well being	Mental well-being: unemployment	2	17-06-2022
Sleep, Well being	sleep: stress	2	27-10-2022
Well being	stress: be in control	5	17-05-2022
Well being	stress: family bounds	2	02-09-2022
Well being	stress: headache	1	17-05-2022
Well being	stress: it adds up	1	27-10-2022
Well being	stress: negative health effects	8	17-05-2022
Well being	stress: personality/coping	10	02-09-2022
Well being	stress: physical complaints	3	17-05-2022
Well being	stress: relaxation	5	02-09-2022
Well being	stress: time restraints	2	02-09-2022
Well being	stress: well-being	5	10-05-2022
Well being	stress: work	9	24-05-2022
Well being	well-being: general	1	03-06-2022

Groups	Name	Groundedness	Creation Date
	healthy lifestyle: social participation	6	05-09-2022
	healthy lifestyle: stay active	7	17-06-2022
